# Following the curvature: Sulcal versus gyral cortical localization of cerebral microbleeds in cerebral amyloid angiopathy

**DOI:** 10.1177/0271678X261482016

**Published:** 2026-08-19

**Authors:** Manon R Schipper, Thijs W van Harten, Emma A Koemans, Jelle J Goeman, Marieke JH Wermer, Matthias JP van Osch, Marianne AA van Walderveen

**Affiliations:** 1 Department of Radiology, Leiden University Medical Center, Leiden, The Netherlands; 2 Department of Neurology, Massachusetts General Hospital, Harvard Medical School, Boston, MA, USA; 3 Department of Neurology, Leiden University Medical Center, Leiden, The Netherlands; 4 Department of Biomedical Data Sciences, Leiden University Medical Center, Leiden, The Netherlands; 5 Department of Neurology, University Medical Center Groningen, Groningen, The Netherlands

**Keywords:** Cerebral amyloid angiopathy, Dutch-type hereditary cerebral amyloid angiopathy, cerebral microbleeds, cortical curvature, inflammation, CSF flow

## Abstract

Cerebrospinal fluid (CSF) mediated brain clearance and inflammation are known features of cerebral amyloid angiopathy (CAA), that may be affected by the cortical gyrencephalic structure. Considering these processes in the development of cerebral microbleeds (CMB), we compared sulcal and gyral cortical CMB densities in sporadic and Dutch-type hereditary CAA (sCAA and D-CAA). CMBs were annotated on 3-Tesla susceptibility weighted imaging. Sulcal and gyral cortex-maps were created using FreeSurfer’s curvature analysis. Sulcal and gyral CMB densities were compared using a generalized linear mixed model. Sensitivity analyses examined sulcal versus gyral CMB densities (1) after adjusting for disease type (sCAA vs D-CAA) and (2) in participants with lobar and deep CMBs. Thirty-nine participants were included (28/11 sCAA/D-CAA, mean age 70/53 years). Sulcal CMB density was higher than gyral CMB density (*B* = 0.57, exp(*B*) = 1.76, *p* < 0.001, exp(*B*) provides the CMB density ratio_(sulcal vs gyral)_). Sensitivity analyses showed robustness of sulcal CMB predominance when taking disease type into account (*B* = 0.46, exp(*B*) = 1.59, *p* = 0.036), with higher densities in sCAA (*B* = 1.36, exp(*B*) = 3.90, *p* = 0.045), and when analyzing the participants with lobar and deep CMBs (*B* = 0.62, exp(*B*) = 1.86, *p* < 0.001). Sulcal CMB predominance in CAA may indicate varying hemorrhagic vulnerability along the cortex, potentially arising from anatomy-based inflammatory and CSF flow dynamics variations.

## Graphical abstract

**Graphical Abstract:**
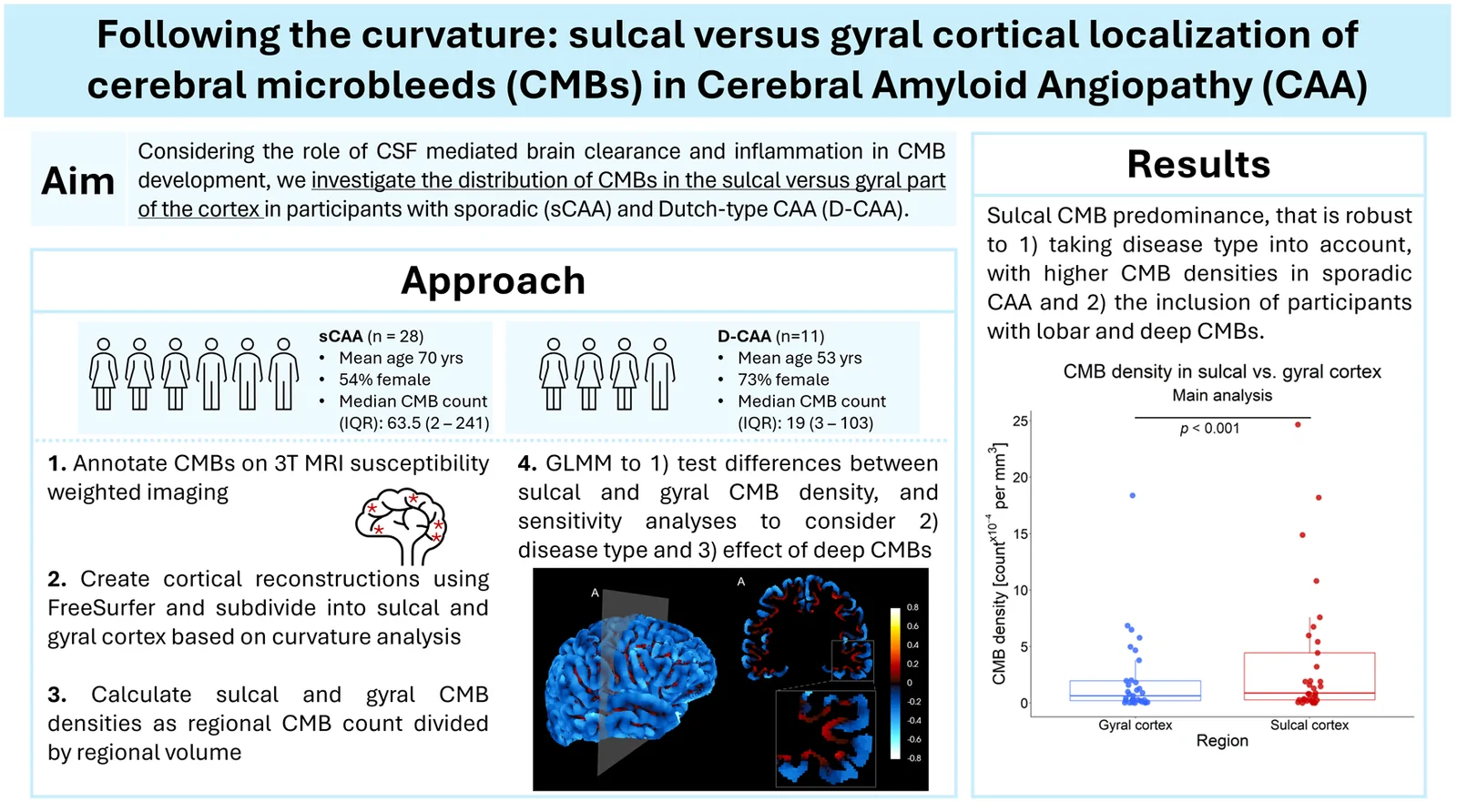
This is a visual representation of the abstract.

## Introduction

Cerebral amyloid angiopathy (CAA) and its monogenetic variant Dutch-type CAA (D-CAA) are cerebral small vessel diseases (cSVD) that are characterized by amyloid-β (Aβ) deposition along the walls of leptomeningeal and cortical arteries.^1^ CAA is one of the leading etiologies of intracerebral hemorrhage (ICH) in the elderly and has a high prevalence of cerebral microbleeds (CMBs), amongst other cSVD related MRI markers. Impaired Aβ-clearance is the leading hypothesis for the underlying disease mechanism.^2^ The underlying pathology of D-CAA is very similar to that of sporadic CAA (sCAA), however, disease onset occurs earlier and progression is more severe. This allows us to study a relatively “pure” form of CAA, with limited presence of age-related vascular risk factors.

Spatial location and topographical distribution of cSVD markers are of importance for diagnosis and may contribute to the understanding of underlying disease mechanisms. For example, CAA is primarily associated with lobar CMBs and, to a lesser degree, cerebellar CMBs, whereas hypertensive vasculopathy is associated with deep (basal ganglia, thalamus, and brainstem) and cerebellar CMBs.^3–5^ Moreover, prior studies have identified a posterior predilection of CMBs in CAA.^6^ As identified with ultra-high field 7 Tesla (7 T) MRI, most CMBs in CAA are located cortically, only a very small portion is located juxtacortical.^7^ PET imaging and histopathological assessment of CAA severity demonstrate its correlation to CMB burden.^8,9^ Interestingly, histopathological research has shown that vessels located at the core of individual CMBs often show reduced Aβ accumulation, likely due to vascular remodeling. Reactive astrocytes and activated microglia are commonly observed surrounding the remodeled vessels,^9^ suggesting the role of inflammation in the occurrence of hemorrhagic events.

In multiple sclerosis (MS), a chronic neuroinflammatory and neurogenerative disease of the central nervous system, cortical lesions develop preferentially in the sulcal cortex rather than in the gyral cortex.^10,11^ Its association with inflammatory cerebrospinal fluid (CSF)-mediated lesion pathogenesis has been suggested.^10,12^ In addition, impaired brain clearance has been hypothesized to play a role in MS, with supporting results of redistributed aquaporin-4 water channels and delayed and prolonged MRI contrast enhancement in meningeal lymphatic vessels in MS patients.^13–15^ Similar processes, for example, inflammation and CSF-mediated brain clearance, play a role in CAA and are related to the development of CMBs.^16–20^

To increase our understanding of mechanisms related to CMB occurrence, we explore whether there is a difference in CMB distribution in the sulcal versus the gyral regions of the cortex (referred to as sulcal and gyral CMB in the remainder of the paper) in sCAA and D-CAA.

## Methods

### Study design and participants

Patients with sCAA and D-CAA and pre-symptomatic D-CAA mutation carriers were retrieved from the prospective natural history study on D-CAA (AURORA) and sCAA (FOCAS). D-CAA diagnosis was based on a DNA-proven APP mutation and sCAA diagnosis was based on the modified Boston criteria—typically assessed on previously acquired clinical scans. Inclusion criteria for both studies are described in detail in previous literature.^21^ For the current study, we included D-CAA and sCAA participants with 3 T MRI T1-weighted and susceptibility weighted imaging (SWI) available, with ⩾1 CMBs, and without ICH. In addition, accuracy of FreeSurfer cortical reconstructions and registrations, as performed in the pipeline described below, were visually checked. Participants were excluded when cortical reconstructions or registrations were inaccurate. Cortical reconstructions were considered as accurate when gray and white matter boundaries represented underlying anatomy and had smooth and continuous surfaces aligning with the T1-weighted imaging. Registrations were considered as accurate when there was complete overlap of anatomical landmarks and precise alignment of anatomical boundaries between registered scans. Participants with ICH were excluded for the current study as ICH typically affects the cortical structure. Scan protocols were identical for participants from the AURORA and FOCAS study.

The AURORA and FOCAS study were executed in accordance with the Helsinki protocol and approved by the Medical Ethics Committee Leiden The Hague Delft and written informed consent was obtained from all participants before enrollment.

### MRI acquisition

MRI scans were acquired on a whole-body 3 T magnetic resonance system (Philips Achieva, Best, The Netherlands) with a standard 32-channel head coil. Scans used in the current study are: three-dimensional T1-weighted images (repetition time (TR)/echo time (TE) = 9/4.6 ms, flip angle (FA) = 8°, 140 slices, and field of view (FOV) = 224 × 178.5 × 168 mm with a voxel size of 1.17 × 1.17 × 1.20 mm) and SWI (TR/TE = 45/31 ms, FA = 13°, 140 slices with no interslice gap, and FOV = 250 × 175 × 112 mm with a voxel size of 0.78 × 0.78 × 1.60, resulting in a scan duration of ~ 6 min).

### Radiological assessment

CMBs were defined—according to the STRIVE criteria 2.0—as signal void areas with associated blooming artifacts on SWI and ⩽10 mm in size, also including non-homogenous lesions.^22–24^ CMBs markers were manually annotated by an observer with over 5 years of experience in the field (EAK), in case of doubt, findings were discussed with a neuroradiologist with over 20 years of experience in the field of neuroradiology (MAAvW). The CMB annotations resulted in a map, containing the coordinates for each CMB.

**Figure 1.**
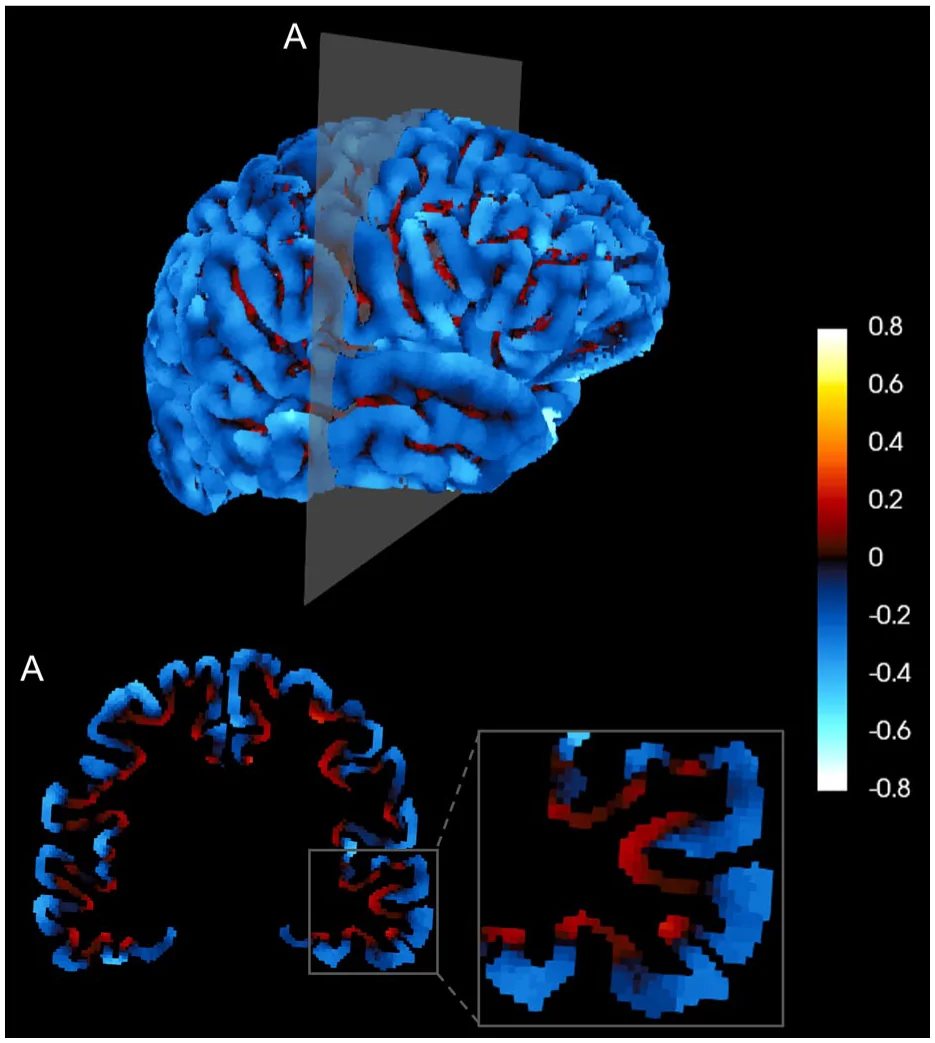
Visualization of curvature values across the cortex as volume rendering (top) and coronal slice with inset (bottom). Positive values (red scale) indicate sulcal regions and negative values (blue scale) indicate gyral regions. Images are rescaled from −0.8 to 0.8 for visualization purposes.

The pipeline to assess the location of CMBs consisted of the following steps: (1) FreeSurfer’s recon-all was run on the T1-weighted images to obtain pial curvature values and cortical reconstructions (FreeSurfer v6.0.0; http://surfer.nmr.mgh.harvard.edu)^25–28^; (2) pial curvature values were projected onto the cortical reconstruction to create cortical curvature maps; (3) cortical curvature maps and atlases of the cerebellum (based on MNI structural atlases)^29,30^ and deep gray matter (GM) structures (based on the Harvard–Oxford atlas)^25,31–33^ were registered to SWI-space per participant based on mutual information registration^34^; (4) cortical curvature maps were divided into sulcal and gyral cortex, based on positive (= sulcal) and negative (= gyral) curvature values. An example of a curvature map is presented in Figure 1; (5) CMB location was identified based on CMB marker overlap with the sulcal or gyral cortex, cerebellum, or deep GM masks. In absence of exact overlap between the marker and cortical mask—and thus non-zero Euclidean distances to all regions—the location of the smallest Euclidean distance was determined and visually confirmed; and (6) volumes of the sulcal and gyral cortex were extracted from the masks. Cerebellar and deep GM atlases were utilized to identify the location of deep CMBs. Steps 3–6 from the pipeline were performed using an in-house developed tool in MeVisLab.^35^

### Statistical analysis

To test the difference between sulcal and gyral CMB density—accounting for regional volume—we performed a Poisson based generalized linear mixed-effects model (GLMM) fit by maximum likelihood (Laplace approximation), using R’s lme4 package.^36,37^ For this main analysis, we included participants with strictly cortical CMBs (allowing cerebellar CMBs). The outcome variable was the raw CMB count, and the model included:

- *Fixed effect:* Cortical region (sulcal vs gyral cortex).
- *Random effects:* A random intercept and slope for cortical region (sulcal vs gyral cortex) per subject, to account for individual variability in CMB density across cortical regions. In addition, the random regional effect handles overdispersion in the data and this overdispersion term allows for adequate modeling of excess zeroes.
- *Offset term:* The log-transformed regional volume to account for varying regional volumes and to model CMB density (CMBs per mm^3^). Also, this approach allows us to adjust for increased uncertainties that are introduced by low CMB counts.

The parameter of interest from the GLMM is exp(*B*), which represents the relative CMB density (sulcal vs gyral cortex). Specifically, exp(*B*) is the ratio of CMB densities between sulcal versus the gyral cortex, calculated as:

$$CMB\;density=\frac{CMB\;count}{regional\;volume\;(mm^{3})}$$

If the main GLMM shows a significant difference between CMB densities per cortical region, we perform sensitivity analyses to investigate the robustness of this effect. Specifically, we 1) included an interaction term between cortical region (sulcal vs gyral cortex) and disease type (sCAA vs D-CAA), to assess whether the effect of cortical region on CMB density varies by disease type and 2) reperformed the main analysis in participants with lobar and deep CMBs to investigate if mixed-pathology affects our findings.

All statistical analyses were performed using R v4.3.3,^36^ with α set at 0.05.

## Results

Twenty-eight sCAA participants (mean age 70 years, 54% female) and 11 D-CAA participants (mean age 53 years, 73% female) were included (Supplemental Figure 1). Additional characteristics and measures are provided in Table 1. Six participants (100% sCAA) had CMBs located in the deep GM or WM. The participants with deep CMBs were excluded from the main analysis and the first sensitivity analysis.

**Table 1.** Baseline characteristics and CMB descriptives of participants with lobar and deep CMBs.

Characteristics	sCAA (*n* = 28)	D-CAA (*n* = 11)
Mean age ± SD (years; range)	70 ± 7 (56–84)	53 ± 7 (45–68)
Female sex (%)	15 (54)	8 (73)
Median total CMB count (IQR)	64 (2–241)	19 (3–103)
Sulcal CMB count	27 (11–101)	3 (1–30)
Gyral CMB count	36 (12–105)	4 (2–65)
Cerebellar CMB count	1 (0–2)	1 (0–10)
Deep GM CMB count	0 (0–0)	0 (0–0)
WM CMB count	0 (0–0)	0 (0–0)
Mean volume sulcal cortex ± SD (cm^3^; range)	190 ± 24 (148–234)	188 ± 16 (164–213)
Mean volume gyral cortex ± SD (cm^3^; range)	460 ± 49 (392–556)	477 ± 48 (410–557)
Median sulcal CMB density (number per mm^3^; IQR)	14.4 × 10^−5^ (5.9 × 10^−5^–50.3 × 10^−5^)	1.7 × 10^−5^ (0.2 × 10^−5^–16.8 × 10^−5^)
Median gyral CMB density (number per mm^3^; IQR)	8.3 × 10^−5^ (2.6 × 10^−5^–21.0 × 10^−5^)	0.9 × 10^−5^ (0.3 × 10^−5^–14.3 × 10^−5^)
Hypertension (%)	17 (61)	3 (27)
Diabetes mellitus (%)	1 (4)	1 (9)
Hypercholesterolemia (%)^a^	11 (39)	1 (9)
Smoking^b^		
Never (%)	6 (21)	4 (36)
Ever (%)	4 (14)	4 (36)
Current (%)	11 (39)	3 (27)
First clinical presentation^c^		
Genetic testing (%)	NA	11 (100%)
TFNE (%)	6 (21)	0 (0%)
Hemorrhage (%)	2 (7)	0 (0%)
Cognitive decline (%)	4 (14)	0 (0%)
Other (%)	6 (21)	0 (0%)

D-CAA: Dutch-type cerebral amyloid angiopathy; sCAA: sporadic cerebral amyloid angiopathy; CMB: cerebral microbleeds; WM: white matter; cSVD: cerebral small vessel disease; TFNE: transient focal neurological episode; NA: not applicable.

a One missing value for hypercholesterolemia in the sCAA group.

b Seven missing values for smoking in the sCAA group.

c Ten missing values for first clinical presentation in the sCAA group. First clinical presentation was categorized as “other” when sCAA was discovered while the patient had non-sCAA related complaints. The cases with hemorrhage as first clinical presentation, were no disruptive ICHs.

**Figure 2.**
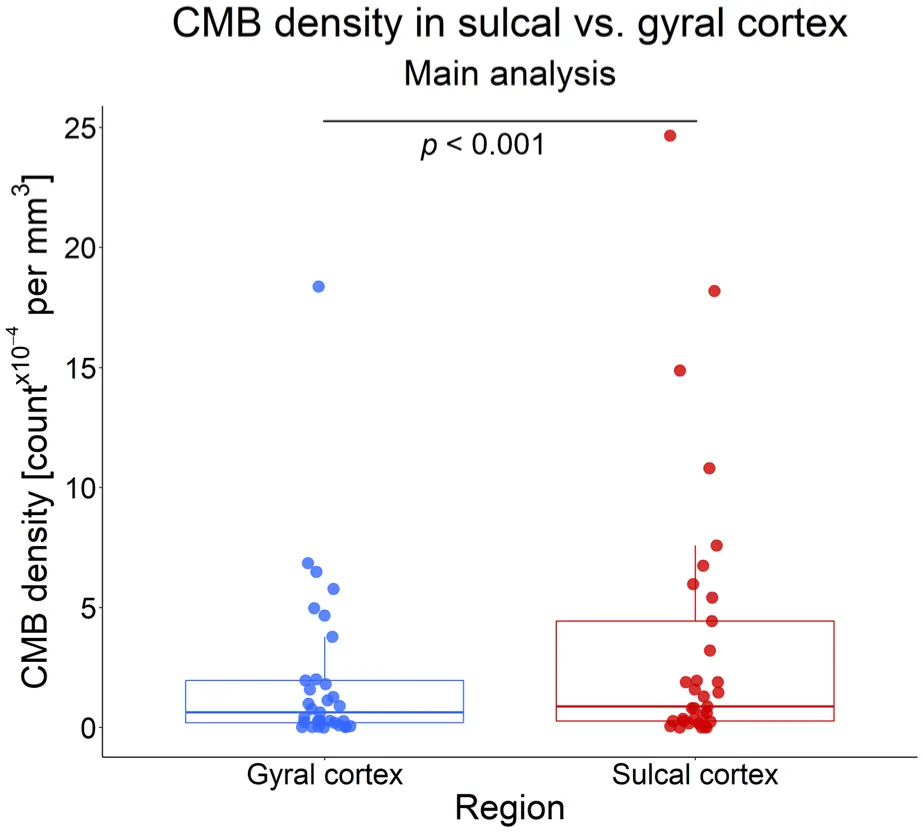
Boxplots of CMB density for both sulcal and gyral cortex for participants with strictly cortical CMBs. CMB: cerebral microbleeds.

Poisson GLMM to assess regional CMB density differences, showed a higher sulcal CMB density (median = 8.76 × 10^−5^, IQR = 2.65 × 10^−5^–44.36 × 10^−5^) compared to gyral CMB density (median = 6.27 × 10^−5^, IQR = 1.98 × 10^−5^–19.58 × 10^−5^; *B* = 0.56, 95% CI (*B*) = 0.33–0.80, SE = 0.11, exp(*B*) = 1.76, 95% CI (exp(*B*)) = 1.39–2.22, *p* < 0.001; Table 2 and Figure 2).

**Table 2.** Statistics from the main generalized linear mixed-effects model testing the distribution of the CMB density in the sulcal versus gyral cortex.

Random and fixed effects	Main analysis			
	*B*	SE	Exp(*B*): relative CMB density	*p*-value
Fixed effects				
Cortical region: sulcal cortex (vs gyral cortex)	0.57	0.11	1.76	<0.001
	Variance	SD		Correlation
Random effects				
Individual variation per region	3.61	1.90		
Cortical region: sulcal cortex (vs gyral cortex)	0.16	0.40		−0.07
	Sensitivity analysis one—including disease type			
	*B*	SE	Exp(*B*): relative CMB density	*p*-value
Fixed effects				
Cortical region: sulcal cortex (vs gyral cortex)	0.46	0.22	1.59	0.036
Disease type: sCAA (vs D-CAA)	1.36	0.68	3.90	0.045
Cortical region × disease type	0.13	0.23	1.14	0.561
	Variance	SD		Correlation
Random effects				
Individual variation per region	3.00	1.79		
Cortical region: sulcal cortex (vs gyral cortex)	0.16	0.40		−0.08
	Sensitivity analysis two—participants with lobar and deep CMBs			
	*B*	SE	Exp(*B*): relative CMB density	*p*-value
Fixed effects				
Cortical region: sulcal cortex (vs gyral cortex)	0.62	0.10	1.86	<0.001
	Variance	SD		Correlation
Random effects				
Individual variation per region	3.23	1.80		
Cortical region: sulcal cortex (vs gyral cortex)	0.15	0.39		−0.15

CMB: cerebral microbleeds; *B*: unstandardized regression coefficient; SE: standard error; exp(*B*): exponent of the unstandardized regression coefficient; SD: standard deviation; sCAA: sporadic Cerebral Amyloid Angiopathy; D-CAA: Dutch-type Cerebral Amyloid Angiopathy.

**Figure 3.**
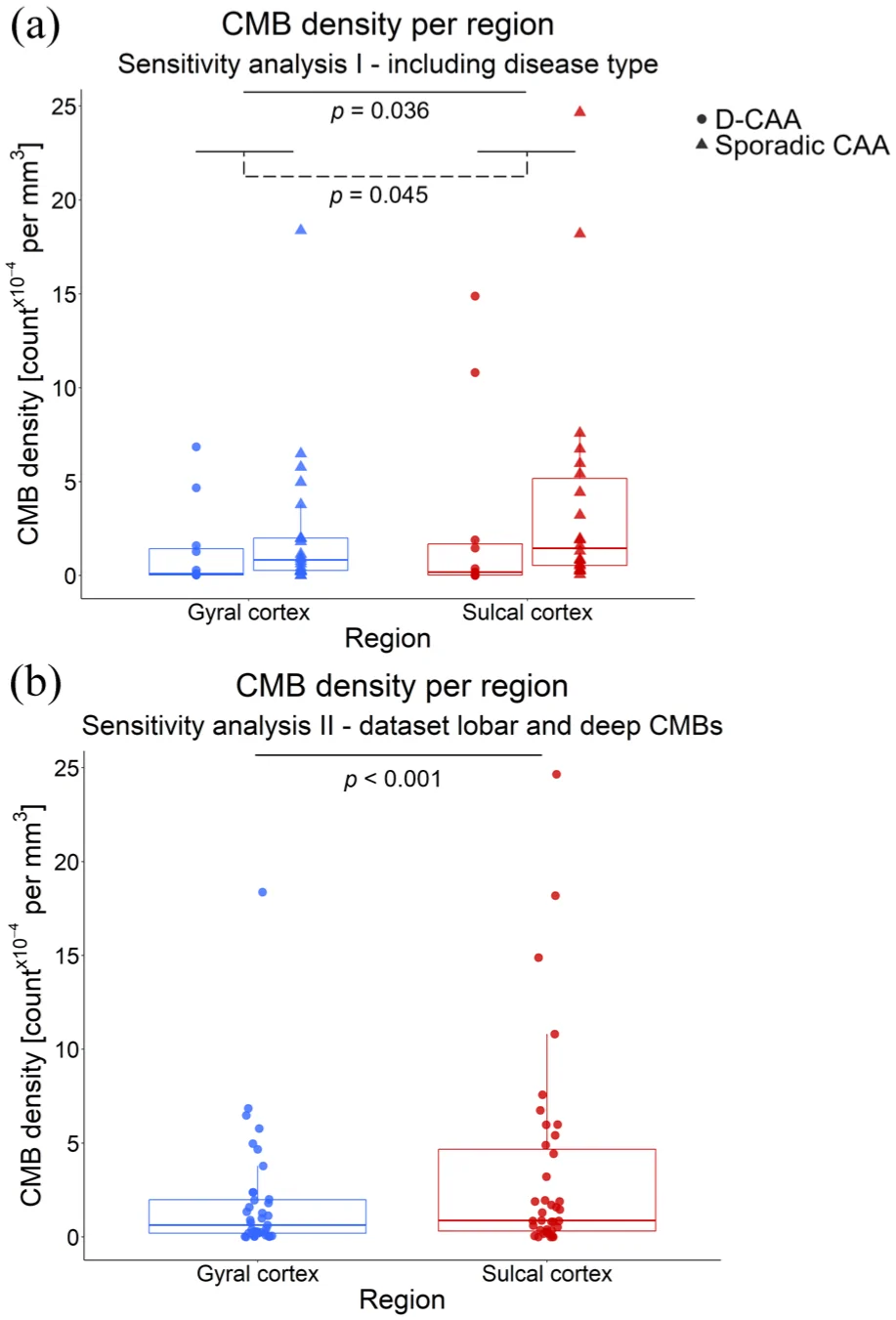
Boxplots for the sensitivity analyses: (a) CMB density for both sulcal and gyral cortex with a subdivision on disease type and (b) CMB density for both sulcal and gyral cortex for participants with lobar and deep CMBs. CMB: cerebral microbleeds; D-CAA: Dutch-type cerebral amyloid angiopathy; CAA: cerebral amyloid angiopathy.

In the first sensitivity analysis, that incorporates disease type, the regional difference in CMB density—that is, higher sulcal CMB density—remained (*B* = 0.46, 95% CI (*B*) = −0.01 to 0.92, SE = 0.22, exp(*B*) = 1.59, 95% CI (exp(*B*)) = 0.99–2.49, *p* = 0.036), and additionally showed a significant effect of disease type with higher CMB densities in sCAA (*B* = 1.36, 95% CI (*B*) = 0.002–2.75, SE = 0.68, exp(*B*) = 3.90, 95% CI (exp(*B*)) = 1.00–15.71, *p* = 0.045; Tables 1 and 2 and Figure 3(a)).

The second sensitivity analysis, that assesses the regional difference in CMB density in the participants with lobar and deep CMBs, showed consistent results of increased sulcal CMB density (median = 8.76 × 10^−5^, IQR = 2.92 × 10^−5^–47.82 × 10^−5^) compared to gyral CMB density (median = 6.27 × 10^−5^, IQR = 2.00 × 10^−5^–19.93 × 10^−5^; *B* = 0.62, 95% CI (*B*) = 0.42–0.82, SE = 0.10, exp(*B*) = 1.86, 95% CI (exp(*B*)) = 1.52–2.27, *p* < 0.001; Table 2, Figure 3(b), and Supplemental Figure 2).

## Discussion

We found a higher CMB density in the sulcal cortex as compared to the gyral cortex. Sensitivity analyses have shown that this effect remains robust when incorporating the effect of disease type (D-CAA vs sCAA), with higher sulcal and gyral CMB densities in participants with sCAA, and when analyzing the participants with lobar and deep CMBs.

CMB distribution along the cortex and its increased sulcal density are likely the result of a complex interplay of multiple processes, including inflammation and CSF flow. Inflammatory processes—for example, leptomeningeal enhancement and CSF non-nulling on FLAIR imaging—are associated with CAA-related inflammation (CAA-ri) and CAA, respectively.^16–19,38^ When hypothesizing that inflammation varies along the cortex, this could be an important explanation of our findings. Such spatial variation might also be explained by CSF flow patterns that will be different along sulci compared to gyri.^39^ Altered CSF flow has been linked to Aβ accumulation in Alzheimer’s disease and its relationship with inflammation has previously been suggested.^10,40–42^ More specifically, the influence of CSF flow on CMB occurrence, through its two-way association with clearance and inflammation, may be hypothesized.^43^ Factors that may influence CSF flow properties are morphology, arterial pulsations, aquaporin-4 water channels, and potentially membranes in the subarachnoid space.^40,44–46^ Although translation of non-human imaging to human research is difficult, imaging in ferrets has shown increased tracer signals in the superficial parts of sulci, indicating efficient CSF influx from the superficial subarachnoid space—hinting towards morphological effects on CSF flow.^47^

The increased sulcal CMB densities in both sCAA and D-CAA separately, show the robustness of our findings. Increased CMB density observed in sCAA compared to D-CAA is likely influenced by our exclusion criteria of having a symptomatic ICH. Shorter disease duration until symptomatic ICH onset in D-CAA, which is around 54 years of age, would explain the density differences between sCAA and D-CAA.^48,49^

Even though patients with sCAA were included into the study based on probable CAA diagnosis according to the modified Boston criteria, retrospectively we noticed a subset of participants with CMBs located in the WM and deep GM. These retrospectively noticed deep CMBs are likely due to the advanced quality of the 3 T research scans as compared to the previously acquired (typically 1.5 T) clinical scans. We performed the main analysis in participants with strictly cortical CMBs. To assess the robustness of our results, we performed a sensitivity analysis to assess the effect in participants with lobar and deep CMBs. The results showed robustness of our main finding, hinting towards a negligible effect of other pathologies on the cortical CMB distribution in the current sample.

Strengths of the current study are that we included participants with a relatively “pure” form of CAA through the D-CAA sample, who have limited age-related co-pathologies resulting in a subset without WM or deep GM CMBs. In addition, our cohort includes a heterogenous group with substantial variability in CMB count; ranging from one, up to 1244. The inclusion of individuals with such high CMB counts enhances our statistical power, allowing for robust estimation of regional differences in CMB density. Also, although the use of the modified Boston criteria in combination with our inclusion criteria of ⩾1 CMB, might have inherently led to exclusion of some very early-stage and very late-stage cases, the wide variability of CMB counts and age within our cohort suggests that our sample represents a broad spectrum of CAA severity.

In the current analyses, we did not explicitly correct for age-related atrophy. Cortical atrophy reduces regional brain volume, which could potentially lead to an overestimation of CMB density, particularly if one region thins faster than the other. Previous research has demonstrated that age-related sulcal thinning is more pronounced than gyral thinning.^50^ However, Table 1 shows similar regional volumes for D-CAA and sCAA. Furthermore, we have illustrated stability of sulcal volumetric measurements by plotting regional volumes as function of age (Supplemental Figure 3), this suggests that cortical thinning likely does not significantly influence our results. Lastly, while cerebellar CMBs were present in some participants, this study focused on supratentorial CMBs, since the curvature analysis cannot be applied to the cerebellum, therefore preventing us from accurately identifying the sulcal and gyral cortex in the cerebellum.

In conclusion, this study demonstrates higher CMB density in sulcal cortical regions in participants with sCAA and D-CAA. To gain further insights on mechanisms underlying these regional differences, future research should explore CSF flow dynamics—particularly in sulcal and gyral regions—in relation to CMB distribution and investigate inflammatory vulnerability along the cortex, for example, through histopathological analyses.

## Supplemental Material

sj-docx-1-jcb-10.1177_0271678X261482016 – Supplemental material for Following the curvature: Sulcal versus gyral cortical localization of cerebral microbleeds in cerebral amyloid angiopathy Supplemental material, sj-docx-1-jcb-10.1177_0271678X261482016 for Following the curvature: Sulcal versus gyral cortical localization of cerebral microbleeds in cerebral amyloid angiopathy by Manon R Schipper, Thijs W van Harten, Emma A Koemans, Jelle J Goeman, Marieke JH Wermer, Matthias JP van Osch and Marianne AA van Walderveen in Journal of Cerebral Blood Flow & Metabolism
