# Supplementary material for: Following the curvature: Sulcal versus gyral cortical localization of cerebral microbleeds in cerebral amyloid angiopathy
Source: J Cereb Blood Flow Metab. 2026 Aug 19:0271678X261482016. Online ahead of print. doi: 10.1177/0271678X261482016 (PMC13593935; doi:10.1177/0271678X261482016)
Supplement: Supplementary file 1 [file sj-docx-1-jcb-10.1177_0271678X261482016.docx]

**Supplementary Figures**

**Supplementary Figure 1. Inclusion flowchart**

*Abbreviations.* sCAA; sporadic Cerebral Amyloid Angiopathy. D-CAA; Dutch-type Cerebral Amyloid Angiopathy. SWI; susceptibility weighted imaging.

**Supplementary Figure 2.** Scatterplot showing CMB density for the sulcal and gyral cortex for participants with lobar and deep CMBs, with a dashed line indicating the midline.

*Abbreviations.* CMB; cerebral microbleeds. D-CAA; Dutch-type Cerebral Amyloid Angiopathy. CAA; Cerebral Amyloid Angiopathy.

**Supplementary Figure 3.** Scatterplot showing volumes for both the gyral and sulcal cortex as function of age, with a linear regression line per region.

*Abbreviations.* CMB; cerebral microbleeds. D-CAA; Dutch-type Cerebral Amyloid Angiopathy. CAA; Cerebral Amyloid Angiopathy.
